# Patients' Perception of Missed Nursing Care in a Tertiary Hospital: A Cross‐Sectional Study

**DOI:** 10.1002/nop2.70157

**Published:** 2025-02-14

**Authors:** Afia Achiaa Sarpong, Amanda Towell‐Barnard, Lucy Gent, Ebenezer Afrifa‐Yamoah, Diana Arabiat

**Affiliations:** ^1^ School of Nursing and Midwifery Edith Cowan University Perth Australia; ^2^ Centre for Nursing Research Sir Charles Gairdner Hospital Perth Australia; ^3^ Aflac Cancer and Blood Disorders Center Children's Healthcare of Atlanta Atlanta GA USA; ^4^ School of Science Edith Cowan University Perth Australia; ^5^ Maternal and Child Nursing Department, Faculty of Nursing The University of Jordan Amman Jordan

## Abstract

**Aims:**

To identify types of patient‐reported missed nursing care and adverse events and identify the factor associated with missed care in a Western Australian tertiary hospital.

**Background:**

Research in the domain of missed nursing care has established the association between missed nursing care and unsafe patient outcomes. However, there is a paucity of evidence on patient perception of missed nursing care and associated factors.

**Design:**

Cross‐sectional study.

**Method:**

A study of inpatients in a tertiary teaching hospital in Western Australia was carried out. Ninety‐eight patients admitted in 16 medical and surgical wards were sampled. The patient MISSCARE survey was used to capture patient‐reported types of missed care, prevalence of adverse events and the association between missed care, unit type, demographic characteristics and patient health problems. Descriptive and logistic analysis were performed using SPSS.

**Findings:**

The most frequently reported missed nursing care activities included mouth care (63%), ambulation (35%), helping patient out of bed into a chair (31%), informing patient about tests or procedures (29%) and considering patient opinion (27%). The majority of reported missed care activities were from basic care domain followed by communication. The most common adverse events reported were intravenous infusion leaking into skin, intravenous fluid running dry, falls and development of pressure ulcer. Significant association was observed between missed nursing care and patient age.

**Discussion:**

The findings of this study showed significant level of patient‐reported missed nursing care particularly in the domain of basic care. Although this study's limitations, including its small sample size and potential response bias, warrant caution in generalising the findings, the insights gained provide a valuable foundation for future research aimed at improving patient care.

**Conclusion:**

Findings provide valuable insight about patient's perception of missed care and inform the need for effective interventions to reduce unsafe outcomes related to missed nursing care.

**Relevance to Clinical Practice:**

Healthcare systems need to make necessary efforts to ensure that patients receive the right amount of care needed to maintain and promote safe hospitalisation outcomes.

**Reporting Method:**

The study was reported according to the STROBE guidelines.

**Patient or Public Contribution:**

The development of this study protocol, data collection, analysis and interpretation of results were carried out through a collaborative effort between patients, families and the research team.

## 1 Introduction

The engagement of hospitalised patients is critical in the ongoing discourse for health care reforms, as it provides the opportunity for the integration of research evidence on experiences and needs (Forsythe et al. 2019). Knowledge on patients' experiences with healthcare systems is increasingly recognised as being vital for nurse managers, researchers and policy makers. Patient engagement offers varying and complementary perspectives of care delivery and may have limited potential risk of bias compared to health workers engagement in research (Miller et al. 2018).

Consumer engagement in healthcare is not only a requirement for safe outcomes but also consumers' input is important in assessing health services delivery of quality care (Miller et al. 2018). Founded on the principle of health systems' policy, research and healthcare delivery, the patient engagement concept serves public interest by patients playing an important role in contributing to care decisions (Struthers et al. 2019). In a review by Degeling et al. (2015), consumer engagement captured a variety of patient and family perspectives and led to investigation of acceptable approaches to produce evidence for policy making. Evidence suggests that promotion of patient engagement in healthcare supports the quality and clinical relevance of health systems research (Wiles et al. 2022).

That said, there is limited contemporary research that examines patient‐reported prevalence of missed nursing care and associated factors among hospitalised patients (Cho et al. 2017; Kalisch et al. 2014). It is essentially crucial for healthcare providers to identify evidence on patient‐reported missed nursing care and associated factors during hospitalisation to improve care outcomes and reduce or prevent missed care. This paper addresses these knowledge gaps and further provides understanding of patient‐reported types of missed care, adverse events and factors associated with missed.

## 2 Background

Missed nursing care is an increasingly recognised consequence of patient hospitalisation with a reported global prevalence of about 55%–98% (Jones et al. 2015). Ensuring patient safety through the provision of complete and adequate care is a major challenge facing health systems today (Lam et al. 2018). Strong evidence suggests that, over one in 10 hospitalised patients experience harm from failure to maintain safe care and globally, this leads to more than 3 million deaths annually (Slawomirski and Klazinga 2022). In a recent patient safety report by the Organisation for Economic Co‐operation and Development, was estimated that, the burden of unsafe patient care outcomes on health systems was about 64 million Disability‐Adjusted Life Years annually, hence a duty for healthcare providers to protect patients from unsafe care outcomes (Slawomirski and Klazinga 2022). With nurses being the healthcare workers providing the most direct care to patients, they have a critical role to play in detecting errors and eliminating patient harm.

Nursing care is a multifaceted process aimed at delivering safe, ethical and individualised patient care, that address the whole person to achieve the best possible health outcomes (Kalisch et al. 2009; Jones et al. 2015). However, there may be situations where nurses are unable to deliver all aspects of care because of various factors (Jones et al. 2015). As a result, certain aspects of care might be missed, reduced or delayed which can lead to care omissions such as missed ambulation, mouth care, feeding and medication error. Worth noting is the fact that these errors can be a serious threat to patient safety (Jones et al. 2015; Tabatabaee et al. 2022).

Previous studies have demonstrated the prevalence of missed nursing care and associated impact on quality of care (Gustafsson et al. 2020; Kalisch et al. 2014; Kim et al. 2018). This has led to a growing interest in research on missed nursing care which has been used interchangeably with terms such as care left undone, unfinished nursing care (Ausserhofer et al. 2013; Lucero et al. 2009) or rationing of nursing care (Schubert et al. 2008). Missed nursing care is defined as the delay, or omission of any aspect of patient care activity (Kalisch et al. 2009). The term reflects nurses' decision‐making process and prioritisation of patient care amidst inadequate human and material resources. The phenomenon of missed nursing care is a universal health problem and has been studied widely in the United States (Campbell et al. 2020; Villamin et al. 2019), Europe (Eskin Bacaksiz et al. 2020; Kalánková et al. 2022; Uchmanowicz et al. 2020), Australia (Blackman et al. 2020, 2018; Henderson et al. 2016; Sarpong et al. 2023), Asia (Cho et al. 2021; Jeong and Min 2021; Labrague et al. 2022) and some parts of Africa (Basazin Mingude et al. 2022; Gathara et al. 2018; Hammad et al. 2021). These studies among others have examined and demonstrated the consequences of this phenomenon on patient outcomes such as falls, hospital‐acquired infection and pressure injury (Aiken et al. 2018; Chaboyer et al. 2021).

Several studies have reported that, nurses frequently miss elements of patient care such as ambulation, teaching, mouth care, interdisciplinary conferences and mouth care (Albsoul et al. 2019; Lake et al. 2020; Zeleníková et al. 2020). These studies among others have demonstrated the contributing factors and the consequences of this phenomenon on patient outcomes. However, the majority of these studies have focused on nurse‐reported findings (Gustafsson et al. 2020).

In contrast, few studies worldwide have examined patients' perceptions of the types of nursing care missed, factors associated with missed nursing care and the impact on patient outcomes (Cho et al. 2017; Kalisch et al. 2014). Considering the increased emphasis on the significance of consumer engagement in healthcare research, eliciting patient perceptions and experience of missed nursing care is one way to enhance patient‐centred interventions and provide better understanding of patients' needs.

Taken all together, and considering hospital and patients' demographic characteristics, the theoretical framework developed by Kalisch and colleagues, based on the Donabedian's linear model in which structure affects process and process in turn affects outcomes was utilised in this study (Donabedian 1966; Kalisch et al. 2009). This Missed Nursing Care Model proposes a direct relationship between hospital and patient characteristics, (structure), missed nursing care (process) and patient outcomes (such as fall, pressure ulcers and infections). The model highlights the influence of structural factors such as unit type and patient characteristics (including age, education and health status) on processes of nursing care and that leads to missed care, which further negatively influences patient outcomes and results in adverse events.

Guided by the Missed Nursing Care model, this study aimed to explore the following research questions.

1. What is the prevalence of the types of missed nursing care reported by patients in Western Australian acute care settings.
2. What is the prevalence of patient‐reported adverse events (such as fall, skin breakdown/pressure ulcer, medication error and hospital‐acquired infection) in Western Australian acute care settings.
3. What are the demographic and health status factors associated with patient‐reported missed nursing care.

## 3 Methods

### 3.1 Design

A cross‐sectional design was used and the guideline Strengthening the Reporting of Observational Studies in Epidemiology (STROBE) was followed (Data S1).

### 3.2 Participants

The study data were voluntarily collected between July and December 2021 from hospitalised patients admitted into 16 medical and surgical units of a tertiary teaching hospital with over 600 bed capacity in Western Australia. The selection of the patient units was done via a meeting between the researcher and nurse informatics managers of the hospital. The range of patient units included general medicine, nuclear medicine, geriatrics, neurosurgery, orthopaedics, general surgery, cardiothoracic, urology, plastics, ophthalmology, immunology, rheumatology, hepatology, gastroenterology, haematology, oncology and facial trauma. A total of 98 patients from selected medical and surgical wards were include in this study. Inclusion criteria were all hospitalised patients who had received care in the various units for not less than 48 h; aged 18 years and above and proficient in English Language. Patients admitted in intensive care units, maternal care, admitted for mental health problem, patients with coma or cognitive disorders and patients who could not communicate in English were excluded from this study. To satisfy sampling variation across units, patients were recruited from all 16 medical and surgical units. A convenient sampling method was used. Based on Krejcie and Morgan's formular and table for determining sample size (95% confidence interval and 5% error margin), the estimated sample population was 202 (Krejcie and Morgan 1970).

### 3.3 Ethical Considerations

The research protocol obtained approvals from the Human Research Ethics Committee (HREC) of the study hospital on 21 April 2021 (approval number: RGS000004484) and the researcher's university HREC on 4 June 2021 (approval number: 2021‐02580). Following ethical approval and site authorisation, procedures were put in place to begin data collection. Participation was voluntary and each participant consented to partake in the study.

### 3.4 Recruitment

Following ethical approval, the researcher first delivered a face‐to‐face presentation of the study to the hospitals' clinical nurse specialists' group. This was followed by a second virtual presentation (due during the Covid‐19 pandemic) at the nurse educator's forum. The nurse leaders in both presentations expressed interest in the study. Next, an official email was sent to nurse managers of all wards informing them of the commencement of data collection. The researcher then visited each ward with the study packets for distribution. Each envelope contained a printed study flyer, participant information and consent form and survey instrument, all of which were voluntarily distributed to patients admitted in the 16 wards and based on the study inclusion criteria. Participants were informed to contact the researcher with any queries as highlighted in the information sheet. A locked survey collection box designed for the study was allocated to each ward, and participants, their relatives or nurses were informed to deposit completed surveys in the allocated box. Additionally, participants were informed that surveys could be mailed to the researcher at the participant's convenience on discharge. Surveys were completed by patients themselves or with the help of a family member.

To increase participant response in this study, the researcher visited all wards weekly (except on Covid‐19 lockdown periods) to continue to advertise the study, recruit more participants and collect completed surveys.

### 3.5 Measure

The MISSCARE Survey‐Patient was used to measure patient reports of nursing care that was provided or left undone. The tool consisted of 13‐items which assesses how often nurses provided a specific care and how long it took for respondents to receive care across three domains: (1) communication (5 items); (2) basic care (4 items); and (3) timeliness (4 items). For communication items, patients reported on information about knowing their assigned nurse, being listened to, having opinions considered, receiving information about test or procedures and discussing treatment. The items were measured on a 5‐point Likert scale ranging from never (1) to always (5) (Kalisch et al. 2014). Basic care domain items included how often nurses provided mouth care, bathing, helping patient out of bed to sit in a chair and ambulation. These items were measured on a 5‐point Likert scale ranging from never (1) to always (5). The last domain, timeliness measured patient reports of how long it took to receive nursing care such as going to the bathroom, answering call light, responding to call lights and beeping machine or monitors. Items in this domain were measured on a 5‐point Likert scale ranging from < 5 min (1), 5 to 10 min (2), 11 to 20 min (3), 21 to 30 min (4) and more than 30 min (5).

Both communication and basic care items were reverse coded to have higher scores indicating more missed nursing care. The mean score of all 13 items was computed as the overall missed nursing care score. The tool has been used in some patients' reported studies and has proven valid and reliable with a reported Cronbach alpha of 0.834 (Cho et al. 2017; Dabney and Kalisch 2015; Kalisch et al. 2014; Sönmez et al. 2020). The three domains were confirmed using factor analysis (communication [*α* = 0.797]; basic care [*α* = 0.708]; timeliness [*α* = 0.834]) and psychometric properties demonstrated acceptable test–retest reliability, content validity and convergent validity (Dabney and Kalisch 2015).

In rating overall nursing care, participants were asked the question, ‘Overall, how would you rate your nursing care during this hospitalisation?’ This item was measured on a 5‐point Likert scale ranging from poor (1) to excellent (5).

In the adverse event section, participants responded to the question, ‘Did you experience any of the following problems during this hospitalisation?’ The problems included falls, skin breakdown or pressure ulcer, medication error, hospital‐acquired infection and intravenous fluid running dry or leaking into the skin. There was also a category named ‘other problems’ allowing patients to indicate any additional problem experienced during hospitalisation. These items were scored as yes, no or unsure.

Demographics and health problems section of the tool included unit type, age, gender, education, marital status, history of cancer, stroke, lung disease, high blood pressure, diabetes, substance abuse, rheumatoid arthritis and psychiatric problem. The MISSCARE Survey‐Patient has been used internationally and proven valid and reliable (Cronbach alpha coefficient of 0.86), hence has been confirmed psychometrically sound (Cho et al. 2017; Dabney and Kalisch 2015; Kalisch et al. 2014) and accepted as rigorous for use in this study.

### 3.6 Data Analysis

Statistical analyses were performed using SPSS package (International Business Machines Corporation version 29) after data cleaning. Descriptive statistics were performed to visualise the basic information about the data and test of normality was performed to assess data distribution. Missing data were treated as omitted cases and the remaining data were analysed. To evaluate the assumption of normality, we examined residuals from linear regression analysis conducted in SPSS. With a sample size of 98, a visual inspection of the Q–Q plot showed that the residuals generally followed the diagonal line, indicating that the residuals approximated a normal distribution. Minor deviations were observed, but they did not suggest severe departures from normality. The Shapiro–Wilk test was also used to assess normality of the residuals, yielding a *p*‐value of 0.12. This result suggests that the residuals do not significantly deviate from normality at the 0.05 significance level. However, given the small sample size, the test may have limited power to detect deviations from normality. The assumption of homoscedasticity was also assessed to ensure that the residuals have constant variance across levels of the independent variable. The residuals versus fitted values plot did not reveal any clear patterns such as funnelling or systematic changes in spread, suggesting that the variance of residuals is approximately constant across the range of predicted values. A statistical Breusch–Pagan test was further performed to formally assess homoscedasticity. The test resulted in a *p*‐value of 0.27, indicating no significant evidence of heteroscedasticity.

Descriptive analyses were further performed to describe the demographic and health status characteristics using frequencies (%) and means (standard deviation [SD]). Patients' perception of the types and extent of missed nursing care were explored using frequencies. The 13 items measuring missed nursing care were treated dichotomously. Items were considered ‘missed care’ if ‘never’, ‘rarely’ or ‘sometimes’ was selected and ‘not missed care’ if ‘usually’ or ‘always’ was selected. For adverse events, participants who selected unsure were excluded. Due to the small number of cases reported, adverse events were not fit to test for correlation analysis.

Correlation analysis was performed using Spearman rho to identify the relationship between overall missed care and the domains of basic care, communication and timeliness.

Univariate and multivariate regression analyses were performed to explore whether demographic and health status data were associated with patient‐reported missed nursing care. We identified and evaluated relationships based on reported crude and adjusted odds ratios. The predictor variables were tested a priori to verify there was no violation of assumption of no multicollinearity.

## 4 Results

### 4.1 Sample Characteristics

Of the 202 estimated sample size, 98 hospitalised patients' voluntary participated, accounting for a response rate of 49%. The demographic and health characteristics of the participating patients are described in Table 1. Over half of the respondents were admitted in medical units (54%), and the majority had stayed for 5 or more days (48%) in their current hospitalisation. In terms of age, respondents ranged between 18 and 87 with a mean age of 59. There were relatively more males (43%) than females (37%) and transgender (9%). Most respondents completed the survey themselves (*n* = 78, 80%), with the remainder done by a family member. All respondents had some form of education with more than half (52%) having a vocational or higher education. Over half of the respondents (53%) graded their health as good or excellent health. Regarding participants' previously diagnosed health problems, the top three reported health problems were high blood pressure (44%), followed by cancer (22%) and psychiatry problem (21%).

**TABLE 1:** Summary of sample characteristics (*N* = 98).

Characteristics	Label	*n*	%
Unit type	Medical	53	54.1
	Surgical	45	45.9
Length of stay	< 5 days	37	37.7
	5–10 days	31	31.7
	11 or more days	16	16.3
	Missing	14	14.3
Age (years)	Mean (SD)	59.04	18.943
	Range	18–87	
	Young adults (18–64)	42	42.9
	Older adults (65+)	43	43.9
	Missing	13	13.3
Gender	Male	42	42.9
	Female	36	36.7
	Transgender	9	9.2
	Missing	11	11.2
Educational level	High school or less	34	34.7
	Vocational/University or other	51	52.0
	Missing	13	13.3
General health	Poor	13	13.3
	Fair	22	22.4
	Good	35	35.7
	Very good/Excellent	17	17.3
	Missing	11	11.2
Disease history	Cancer	22	22.4
	Lung disease	15	15.3
	Heart isease	18	18.4
	High blood pressure	43	43.9
	Stroke	8	8.2
	Psychiatric problem	21	21.4
	Substance abuse	3	3.1
	Diabetes	14	14.3
	Rheumatoid arthritis	11	11.2

### 4.2 Prevalence and Outcomes of Missed Nursing Care

**FIGURE 1:**
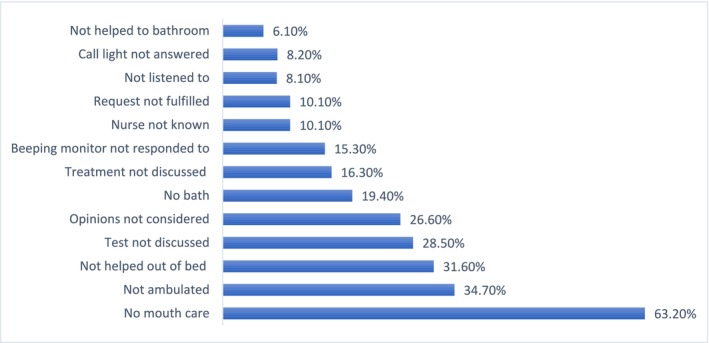
Patient‐reported missed elements of nursing care.

The top three missed elements reported by patient were mouth care (63%) followed by ambulation (35%) and being helped out of bed (32%) (Figure 1). The overall mean missed care (Table 2) reported by patients was 1.88. Of the three domains of missed care, basic care was most missed scoring 2.32, followed by communication at 1.83, with timeliness scoring least at 1.53.

**TABLE 2:** Missed nursing care prevalence by the three domains.

Subscales	*n*	Mean (SD)	Median	Min–Max
Communication	98	1.83 (0.67)	1.60	1.00–4.00
Basic care	97	2.32 (0.98)	2.25	1.00–4.75
Timeliness	97	1.53 (0.56)	1.50	1.00–3.25
Overall missed care	98	1.88 (0.53)	1.81	1.00–4.00

A highly positive and statistically significant result was found between overall missed nursing and basic care (*r = 0*.801, *p* < 0.001) and communication domains (*r = 0*.745, *p* < 0.001). A moderate positive and statistically significant correlation was found between missed nursing care and timeliness (*r = 0*.550, *p* < 0.001).

Patient‐reported adverse events, and overall rating of nursing care are illustrated in Table 3. The highest reported adverse event was intravenous infusion running into skin (9%) followed by fall, pressure ulcer and infusion running dry (6%). Overall, 94% of respondents rated nursing care as good, very good or excellent.

**TABLE 3:** Prevalence of adverse event and rating of nursing care.

Adverse events	*n*	%
Fall		
Yes	6	6.1
No	92	93.9
Skin pressure/Pressure ulcer		
Yes	6	6.1
No	92	93.9
Medication error		
Yes	1	1.0
No	97	99.0
Hospital‐acquired infection		
Yes	4	4.1
No	94	95.9
IV running dry		
Yes	6	6.1
No	92	93.9
IV leaking into skin		
Yes	9	9.2
No	89	90.8
Rating of overall nursing care		
Poor/Fair	3	3.1
Good/Very good or Excellent	92	93.8
Missing	3	3.1

From the binary logistic regression analysis, there were no significant relationship between missed care and demographic characteristics (Table 4) and participants' disease history (Table 5) except for age and gender. The findings that older patients aged 65 years and above were more likely to report missed nursing care were confirmed when adjusted for in multivariate analysis (*p* < 0.030). Regarding gender, there was an association in females and reported missed care compared to males in the *p*‐value of the crude odds (*p* < 0.023), with the effect disappearing in the adjusted analysis (*p* < 0.059) (Table 4).

**TABLE 4:** Binary logistic regression analysis of demographic factors associated with patient‐reported missed nursing care in a tertiary hospital.

Variable	Category	Missed nursing care		Univariable model			Multivariable model		
		Yes	No	Est (se)	Crude odds ratio (95% CI)	*p*	Est (se)	Adjusted odds ratio (95% CI)	*p*
Unit	Medical	41 (77.4%)	12 (22.6%)	0	1	—	0	1	—
	Surgical	32 (72.7%)	12 (27.3%)	0.248 (0.472)	1.281 (0.509, 3.228)	0.599	0.528 (0.654)	1.695 (0.471, 6.102)	0.420
Days in unit	1–5	27 (73.0%)	10 (27.0%)	0	1	—	0	1	—
	5–10	24 (80.0%)	6 (20.0%)	−0.393 (0.588)	0.675 (0.213, 2.136)	0.504	−1.227 (0.729)	0.333 (0.051, 2.158)	0.092
	≥ 11	14 (87.5%)	2 (12.5%)	−0.953 (0.842)	0.386 (0.74, 2.008)	0.258	−1.101 (0.954)	0.092 (0.070, 1.223)	0.249
Age (years)	18–64	36 (87.8%)	5 (12.2%)	0	1	—	0	1	—
	≥ 65	30 (69.8%)	13 (30.2%)	1.138 (0.581)	3.120 (1.098, 9.751)	**0.050**	1.612 (0.741)	4.403 (0.961, 20.171)	**0.030**
Gender	Male	29 (69.0%)	13 (31.0%)	0	1	—	0	1	—
	Female	32 (91.4%)	3 (8.6%)	−1.565 (0.690)	0.209 (0.54, 0.809)	**0.023**	−1.459 (0.771)	0.232 (0.048, 1.121)	0.059
	Transgender	6 (66.7%)	3 (33.3%)	−0.802 (0.334)	1.115 (0.241, 5.164)	0.889	0.935 (0.971)	2.133 (0.269, 16.936)	0.335
Education	≤ High school	27 (79.4%)	7 (20.6%)	0	1	—	0	1	—
	Vocational/University	38 (76.0%)	12 (24.0%)	0.197 (0.538)	1.218 (0.424, 3.497)	0.714	0.781 (0.716)	1.495 (0.324, 6.895)	0.275

*Note:* *p* ≤ 0.05. Missed nursing care (dependent variable) binary scores: 0 = not missed care and 1 = missed care. *p*‐ values in bold italics indicate statistical significance at *p* < 0.05.

**TABLE 5:** Adjusted models estimates for the association of disease history and reported missed nursing care.

Variable	Category	Missed nursing care		Univariable model			Multivariable model		
		Yes	No	Est (se)	Crude odds ratio (95% CI)	*p*	Est (se)	Adjusted odds ratio (95% CI)	*p*
Health status	Poor	11 (84.6%)	2 (15.4%)	0	1		0	1	—
	Fair	20 (90.9%)	2 (9.1%)	−1.428 (1.317)	0.240 (0.018, 3.166)	0.278	−0.446 (1.639)	0.640 (0.026, 15.883)	0.785
	Good	24 (70.6%)	10 (29.4%)	0.604 (0.907)	1.829 (0.309, 10.832)	0.506	1.372 (1.221)	3.944 (0.361, 43.141)	0.261
	Very good	11 (64.7%)	6 (35.3%)	0.849 (0.976)	2.337 (0.345, 15.813)	0.384	1.260 (1.342)	3.527 (0.254, 48.903)	0.347
Cancer	Yes	17 (77.3%)	5 (22.7%)	0.069 (0.668)	1.071 (0.289, 3.965)	0.918	−0.064 (0.866)	0.938 (0.172, 5.128)	0.941
	No	44 (80.0%)	11 (20.0%)	0	1	—	0	1	—
Lung disease	Yes	11 (73.3%)	4 (26.4%)	0.665 (0.756)	1.945 (0.442, 8.555)	0.379	0.699 (1.249)	2.011 (0.174, 23.243)	0.576
	No	50 (78.1%)	14 (21.9%)	0	1	—	0	1	—
Heart disease	Yes	13 (76.5%)	4 (23.5%)	−0.148 (0.770)	0.863 (0.191, 3.889)	0.848	−0.057 (1.098)	0.945 (0.110, 8.120)	0.959
	No	50 (78.1%)	14 (21.9%)	0	1	—	0	1	—
High blood pressure	Yes	31 (73.8%)	11 (26.2%)	0.445 (0.628)	1.560 (0.456, 5.340)	0.479	0.254 (0.789)	1.290 (0.274, 6.066)	0.747
	No	30 (81.1%)	7 (18.9%)	0	1	—	0	1	—
Stroke	Yes	4 (50.0%)	4 (50.0%)	1.387 (0.878)	4.004 (0.716, 22.392)	0.114	0.638 (1.308)	1.892 (0.146, 24.554)	0.626
	No	58 (80.6%)	14 (19.4%)	0	1	—	0	1	—
Psychiatric problem	Yes	18 (85.7%)	3 (14.3%)	−0.212 (0.788)	0.809 (0.173, 3.790)	0.788	0.379 (1.055)	1.461 (0.185, 11.547)	0.719
	No	44 (75.9%)	14 (24.1)	0	1	—	0	1	—
Substance abuse	Yes	3 (100.0%)	0 (0.0%)	−1.605 (1.248)	0.201 (0.012, 5.249)	0.542	−1.396 (1.787)	0.248 (0.024, 5.124)	0.784
	No	59 (77.6%)	17 (22.4%)	0	1	—	0	1	—
Diabetes	Yes	10 (76.9%)	3 (23.1%)	−0.263 (0.913)	0.769 (0.128, 4.601)	0.773	−0.337 (1.148)	0.714 (0.075, 6.775)	0.769
	No	53 (79.1%)	14 (20.9%)	0	1	—	0	1	—
Rheumatoid arthritis	Yes	9 (81.8%)	2 (18.2%)	−1.530 (1.537)	0.222 (0.004, 6.245)	0.645	−1.899 (1.196)	0.150 (0.005, 4.854)	0.845
	No	53 (77.9%)	15 (22.1%)	0	1	—	0	1	—

*Note:* Missed nursing care (dependent variable) binary scores: 0 = not missed care and 1 = missed care.

## 5 Discussion

The results of our study provide valuable insights into patients' perceptions of missed nursing care in Western Australia, highlighting several key areas of reported missed care patient experience. The most frequently reported missed nursing care activities were from the domain of basic care followed by communication. However, areas such as the relationship between missed care, demographic characteristics and adverse events are highlighted as significant areas needing further research. The findings indicate that within this small sample population, there is a modest statistically significant relationship between patient‐reported missed nursing care and age. However, the regression model showed no statistical significance between missed care and all other demographic variables. These findings may be due to the small sample size or other unaccounted factors hence the need for a larger study involving two or more hospitals.

Of note this is the first study in Western Australia that explores patient reported prevalence of missed nursing care, adverse events and the link between missed care, demographic characteristics and disease history. Studying patient perspectives and experiences of missed care and how it impacts health outcomes increases our understanding of the gap in nurse–patient interactions and support the ongoing framework of patient involvement in health systems research (Miller et al. 2018).

This study examined patients' perceptions of missed nursing care and provided empirical evidence quantifying most frequently missed nursing activities, reported adverse events and associated factors. This study highlighted the phenomenon of missed nursing care, an ethical issue of concern challenging the nursing profession; underscoring the need for a clinically safe nursing care environment, to promote safe patient outcomes. The findings of this paper are essential for policy, practice and further research in this domain. Overall, this study revealed that, important patient care activities are missed during hospitalisation.

Within the large tertiary hospital, the top reported missed care activities were mouth care, ambulation, getting patient out of bed into a chair, informing patient about test and procedures, considering patient opinions and ideas and bathing patient. The least patient‐reported activities included help with using the bathroom, answering call light, listening to patient and fulfilling request. Similarly, increased prevalence of missed care were also found in a previous study conducted by Kalisch and colleagues (Kalisch et al. 2014) in two hospitals within the Midwestern region of the United States of America. In their study, the authors investigated patient‐reported missed care and identified mouth care (50%), ambulation (41%), getting patient out of bed (38%), information about test and procedures (27%) and bathing patient (26%) as the five most missed care elements. It is possible that the prevalence of missed care was lower in this study than the previous study on account of the period of data collection (novel coronavirus [Covid‐19] period) and context of data collection. For example, in a study that compared missed care occurrence among Covid‐19 and non‐Covid‐19 patients, nurses concluded that, overall missed care was higher in the pandemic period than pre‐pandemic period (Cengia et al. 2022). The evidence from this study suggests that missed care exist, hence the need for a clear map of action to mitigate its effect on patient outcomes.

Regarding the domains of missed care, patients notably reported the highest missed care in basic care domain followed by communication and lastly timeliness of nursing staff to carry out patient's need. This finding is consistent with a recent review that identified missed volumes of basic care activities (such as mouth care, ambulation, supporting from bed to a chair and bathing) followed by communication activities (such as providing information about test/procedures, discussing treatment plans, considering patient opinion, and informing patient who their nurse was) and timeliness activities (such as timely assistance to bathroom, providing call light request and responding to patient's beeping monitor) (Gustafsson et al. 2020). Failure to provide patient mouthcare can lead to hospital‐acquired pneumonia (Warren et al. 2019) and lack of ambulation have been linked to postoperative delirium (Robinson et al. 2021). Notably, lack of communication has been found to contribute to missed care occurrence in several nurse–patient‐reported studies (Avallin et al. 2020; Chegini et al. 2020; Prosser et al. 2020) and can be an essential tool to address and avoid missed care which has inherent potential for negative hospitalisation outcomes (Avallin et al. 2020).

The significant finding that older adults 65 years and above reported more missed care needs to be further explored and interventions put in place to protect this vulnerable population in acute care settings. Older adults are among the most vulnerable groups with more complex care needs because of multiple chronic conditions. Their vulnerability can contribute to unsafe hospitalisation outcomes such as falls, pressure injuries and hospital‐acquired infections. When deviation from safe and complete care occurs, missed nursing care can have serious consequences on this vulnerable group of patients (Bail and Grealish 2016; Willis and Brady 2021).

The most frequent adverse event reported by participants was intravenous fluid leaking into skin, followed by intravenous fluid running dry, fall and pressure ulcer. The findings from Kalisch and her team also identified similar findings of intravenous fluid leaking into skin and intravenous fluid running dry as the most common adverse events in their study (Kalisch et al. 2014), although their study reported higher levels of these adverse events (15% and 12% respectively). In a more recent study in Turkey, with similar results, higher levels of these events were reported (intravenous fluid leaking into skin 35% and intravenous fluid running dry 40% respectively) (Sönmez et al. 2020). While the proportions of reported adverse events are small, they provide valuable and complementary information about participant's experiences of unsafe outcomes of hospitalisation. For example, in a study of hospital‐acquired complications in South Australia and Victoria, patient characteristics were reported as major determinants of hospital‐acquired complications (Duke et al. 2022). Hence further clinical research is warranted to understand the relationship between patient characteristics and adverse events.

Most of the patient demographic and disease history variables did not influence the likelihood of reporting higher or lower scores of missed nursing care (Tables 4 and 5). Although this study found significant association in age and reported missed care, the associated effect between gender and missed care disappeared in the adjusted odds ratio. Further studies using larger sample size is warranted for better precision and understanding of the relationship between reported missed care and demographic and health history. Worth noting is the fact that, there is limited opportunity for comparing the association between missed care, patient background characteristic and health problem, because few studies have examined patient‐reported missed care and how it relates to patient demographic and disease history. Further, the few studies that do exist have not examined this relationship (Albsoul, FitzGerald and Alshyyab 2022; Cho et al. 2017; Dabney and Kalisch 2015; Kalisch et al. 2014; Sönmez et al. 2020). Nevertheless, compared with the small number of previous studies that have used a validated patient MISSCARE instrument, the overall prevalence of missed care identified in the current study is similar to that reported in previous studies hence the need for desperate measures to assist patients to achieve optimal health (Albsoul, FitzGerald and Alshyyab 2022; Cho et al. 2017; Dabney and Kalisch 2015; Kalisch et al. 2014; Sönmez et al. 2020). These measures can include the use of digital tools such as remote patient monitoring and patient specific portals which can empower patients, improve their understanding of the care process, facilitate better communication with care team and overall, improve care outcomes (Abernethy et al. 2022).

### 5.1 Limitations

Although this study provides important insights into the relationship between age and patient‐reported missed nursing care, one notable limitation is the statistical power of 60%, which is below the commonly recommended threshold of 80%. This lower power increases the likelihood of Type II error, meaning that the study may not have been able to detect smaller, but potentially meaningful relationships between reported missed care and demographic factors. The relatively low power can likely be attributed to the small sample size (98). Although some trends were observed, the lack of sufficient power suggest that these findings should be interpreted with caution. It is possible that with a larger sample size, statistically significant results might emerge. To strengthen the reliability of these findings, future research should aim for a larger sample size to achieve a power of 80% or higher. Increasing the sample size will help ensure that the study is adequately powered to detect smaller effects and provide more robust conclusions regarding the relationship between patient‐reported missed nursing care, demographic factors and reported adverse events.

Another limitation of this study was the fact that it was conducted in a single teaching hospital in Western Australia limiting the generalisability of the findings.

Also, a limitation of the study is the asymmetry of nursing care knowledge and information between patients and care staff. This disparity can affect the quality of care and the accuracy of patient feedback, as patients may not always be fully aware of their care plans, treatment options or the rationale behind certain clinical decisions (Kalisch et al. 2014). Similarly, care staff may not always have complete insights into patient preferences or concerns, which can impact the overall care experience. Future studies can prevent this limitation through implementation of strategies to promote patient communication and schedule regular check‐ins with patients to discuss care plan, address any questions and ensure they are well‐informed about their care and treatment options prior to data collection.

In addition, findings were limited to patient or caregiver reported perceptions of missed care and adverse events which may be under‐reported, over‐reported or lead to response bias. Further studies using observational approaches and health system data may offer better understanding into patient‐reported missed care and related adverse events. Lastly while the majority of the survey were completed by patients themselves, those completed by patient family member may be considered as a limitation because there may have been response bias.

### 5.2 Implications for Nursing Practice

This research has highlighted that nurses currently face challenges in delivering the full scope of care necessary to ensure patient safety. As a result, further research is needed to establish accurate, reliable and objective measures—such as specific missed care activities and the direct impact of each on patient outcomes—that are essential for patient safety, smooth hospital stays, nurse satisfaction and retention. The absence of objective daily measures for reported missed nursing care and its impact on patients is concerning, particularly for complex patients, as it can lead to complications. Utilising the findings from this study as foundational knowledge to guide the development of strategies aimed at reducing patient‐reported missed nursing care may contribute to improved patient outcomes.

## 6 Conclusions

This study found that nurses are not able to meet a large proportion of patient care needs particularly basic care needs. Additionally, inadequate communication in areas such as nurses providing information about test and discussing treatment and timely responses to deliver care in the medical and surgical units were reported. Older adult patients (65 and over) appeared to have higher odds of reporting missed nursing care. Moreover, patients reported the prevalence of adverse events during their current hospitalisation.

Within the limited knowledge about the nature and extent of patient‐reported missed nursing care, these findings inform nurses, managers and healthcare systems about the unmet nursing care elements required by hospitalised patients, and also those aspects of nursing care that may require improvement. Effective strategies and intervention measures are needed to translate this evidence into practice to reduce unsafe patient outcomes.

## Conflicts of Interest

The authors declare no conflicts of interest.

## Supporting information


Data S1.

## Data Availability

Data that support the findings of this study are available on request from the corresponding author. The data are not publicly available due to privacy or ethical restrictions.
